# CD36-mediated ferroptosis destabilizes CD4^+^ T cell homeostasis in acute Stanford type-A aortic dissection

**DOI:** 10.1038/s41419-024-07022-9

**Published:** 2024-09-12

**Authors:** Hui Li, Peng-Fei Wang, Wei Luo, Di Fu, Wei-Yun Shen, Yan-Ling Zhang, Shuai Zhao, Ru-Ping Dai

**Affiliations:** 1 Department of Anesthesiology, the Second XiangYa Hospital, Central South University, ChangSha, China; 2 Anesthesiology Research Institute of Central South University, ChangSha, China; 3 Department of Anesthesiology, XiangYa Hospital, Central South University, ChangSha, China

**Keywords:** Immune cell death, Aortic diseases

## Abstract

Acute type A aortic dissection (ATAAD) is a lethal pathological process within the aorta with high mortality and morbidity. T lymphocytes are perturbed and implicated in the clinical outcome of ATAAD, but the exact characteristics of T cell phenotype and its underlying mechanisms in ATAAD remain poorly understood. Here we report that CD4^+^ T cells from ATAAD patients presented with a hypofunctional phenotype that was correlated with poor outcomes. Whole transcriptome profiles showed that ferroptosis and lipid binding pathways were enriched in CD4^+^ T cells. Inhibiting ferroptosis or reducing intrinsic reactive oxygen species limited CD4^+^ T cell dysfunction. Mechanistically, CD36 was elevated in CD4^+^ T cells, whose blockade effectively alleviated palmitic acid-induced ferroptosis and CD4^+^ T cell hypofunction. Therefore, targeting the CD36-ferroptosis pathway to restore the functions of CD4^+^ T cells is a promising therapeutic strategy to improve clinical outcomes in ATAAD patients.

## Introduction

Acute aortic dissection (AAD) is a life-threatening disease with an incidence ranging from 4 to 7.7 per 100,000 patient-years [1]. AAD is characterized by a tear in the aortic wall that allows blood to flow between the layers of the aorta wall, leading to its potential rupture [2, 3]. AAD has been divided into two types, type-A (ATAAD) and type B, according to whether the ascending aorta was involved or not as defined by the Stanford system. The onset of ATAAD is urgent and unpredictable, with immediate open surgical repair as the sole effective treatment. Despite significant advances in surgical techniques, the in-hospital mortality after ATAAD surgery remains between 17% and 26% [4, 5]. This alarming statistic emphasizes the importance of understanding the factors contributing to its high morbidity and mortality rates, beyond the surgical intervention itself. Addressing these factors will be crucial to improve perioperative care and outcomes for ATAAD patients.

Damaged aortic cells release numerous ‘danger’ molecules after aortic wall rupture, triggering intense responses in the innate immune system, as shown by the high concentrations of inflammatory factors in AAD patients [6]. However, the involvement of the adaptive immune system in AAD and the host’s response to acute aortic injury remain poorly understood. Previous studies have shown changes in Th1, Th2, Th17 and Treg lymphocyte populations associated with the onset of AAD, suggesting a disturbance in T cell responses [7, 8]. Our recent study also revealed a high incidence of lymphopenia in ATAAD and demonstrated that CD4^+^ T cell lymphopenia was associated with poor postoperative outcomes in these patients [9]. Lymphopenia is a hallmark of immunosuppression in sepsis which is often present in patients admitted to the intensive care unit (ICU) and is strongly associated with secondary infections and mortality [10]. These findings lead us to speculate that acute aortic injury may alter the phenotypes and functions of CD4^+^ T cells, which are subsequently implicated in the postoperative outcomes of patients with ATAAD.

Ferroptosis is a well-known non-apoptotic type of programmed cell death that is dependent on intracellular iron [11, 12]. Conceptually, ferroptosis can be considered as a byproduct of cellular metabolism. It results from an overload of iron, an essential driver of metabolism, leading to excessive reactive oxygen species (ROS) production and oxidative modification of lipids in membranes driving the development of ferroptosis [11, 12]. Much research has identified a critical role of ferroptosis in various pathological scenarios including cancer, neurodegeneration and tissue ischemia [12, 13]. Whether ferroptosis promotes T cell dysfunction in aortic lesions remains to be unequivocally established.

Here, we unravel a profile of cell-intrinsic activation defects that limited their expansion or differentiation in ATAAD patients. Fatty acids (FAs) uptake by CD36 drives CD4^+^ T cell ferroptosis, which leads to CD4^+^ T cell dysfunction for ATAAD patients. The results have identified a central cellular defect of CD4^+^ T cell function and revealed the central role of the CD36-ferroptosis axis in regulating CD4^+^ T cell dysfunction, which can be targeted to enhance T function and potentially be beneficial to clinical outcomes in patients with ATAAD.

## Materials and methods

### Patients

A cohort of 55 consecutive ATAAD patients who received total arch replacement was prospectively identified and recruited at the time of admission at the Second Xiangya Hospital of Central South University between February 2021 and March 2022. Patients met the American Heart Association revised criteria for diagnosis of ATAAD [14, 15]. The diagnosis of ATAAD was confirmed by computed tomography [14, 15]. All enrolled patients with ATAAD underwent surgery. Exclusion criteria included infectious diseases, autoimmune disorders, cancer, previous aortic surgery, malignant tumors, and use of steroids or non-steroidal anti-inflammatory medicines. Healthy, gender- and age-matched healthy donors (HDs, *n* = 55) were enrolled. The clinical features of volunteers are given in Table S1. All baseline data were provided by one member in our research team, and outcome documents were collected by a different team member blinded to the baseline data. All documentation was analyzed by a third member of the research group.

### Magnetic-activated cell sorting and cell culture

Peripheral blood mononuclear cells (PBMCs) were isolated by density gradient separation from heparinized blood preoperatively. Magnetic-activated cell sorting (MACS) kit (Miltenyi Biotec, catalog no. 130-045-101) was performed to negatively select CD4^+^ T cells from freshly isolated PBMC with purities of typically >94% as confirmed by flow cytometry (Fig. S1). Cells were cultured in RPMI complete medium (10% fetal bovine serum, catalog no. A3160802, 1% streptomycin/penicillin, catalog no. 15140122) in 37°C and 5% CO_2_, and processed for further experiments. For optimal activation and to achieve an active state, CD4^+^ T cells were stimulated with phytohemagglutinin (PHA) (30 µg/mL) treatment. 50 μM of FAs, palmitic acid (PA), oleic acid (OA) and arachidonic acid (ARA) were used to culture CD4^+^ T cells. The FAs were a mixture of PA, OA and ARA, each at a concentration of 50 μM. To identify the types of cell death, various inhibitors were used in the cell culture, including ferrostatin-1 (Fer-1) (ferroptosis inhibitor, 5 µM), n-acetylcysteine (NAC) (antioxidant, 30 mM), benzyloxycarbonyl-Val-Ala-Asp(OMe)-fluoromethylketone (Z-VAD) (apoptosis inhibitor, 10 µM), and necrostatin-1 (NEC) (necrosis inhibitor, 20 µM).

### Flow cytometry

Cells were labeled with fluorescently-conjugated monoclonal antibodies (mAbs) for 30 min at 4 °C, and then washed with FACS buffer (1% FBS in PBS). Details of staining combinations and reagents are listed in Table S2 and Table S3. Following the addition of counting beads (Biolegend, catalog no. 424902) to determine absolute cell counts, samples were immediately examined using flow cytometry and analyzed with FlowJo software (BD Biosciences). Fresh cells had a viability rate that was consistently > 95%.

### Cell viability quantification

CD4^+^ T cell viability was examined using the Annexin V/7AAD assay with the use of Apoptosis Detection Kit (BD Biosciences, catalog no. 556419, Biolegend, catalog no. 640936). After cell surface staining, cells were analyzed by flow cytometry. Annexin V^−^7AAD^−^ cells were considered to be living cells [16].

### Measurement of mitochondrial function

ROS levels were detected using DCFH-DA (ThermoFisher, 88-5930-74) by FACS. Mitochondrial numbers were assessed using MitoTracker Green (ThermoFisher, M46750) by FACS. Loss of lymphocyte mitochondrial transmembrane potential (ΔΨm) was quantified using JC-1 dye (ThermoFisher, M34152) by FACS.

### RNA-seq analysis

CD4^+^ T cells were isolated from PBMCs and processed with Trizol (ThermoFisher, catalog no. 15596026) to obtain RNA samples. These samples underwent cDNA synthesis and were sequenced using the Genedovo RNA platform. Subsequently, differential expression analysis was conducted with DESeq2, identifying differentially expressed genes (DEGs) with false discovery rate (FDR) < 0.05, absolute fold change ≥ 1.5 and a *P*-value < 0.05. GSEA was employed to determine statistically significant differences in predefined sets of genes between the experimental groups. STRING v10 was used to construct protein-protein interaction networks. Additionally, KEGG pathway and Gene Ontology (GO) analyzes were performed to identify enriched biological pathways and gene functions.

### RNA-seq analysis (public database)

RNA-seq data from GEO dataset GSE190635 were normalized and analyzed. Raw data underwent log2 transformation, and microarray data normalization used the ‘normalize quantiles’ function from the preprocessCore R package (v3.4.1). Probes associated with multiple genes were excluded and expressions averaged for genes with multiple probes. Differential expression, identified using the limma R package, required an adjusted *P*-value < 0.05 and an absolute log2(Fold Change) > 1, visualized using heatmaps from the pheatmap R package. Functional enrichment analysis focused on KEGG pathway enrichment, with pathways significantly enriched at a *P*-value < 0.05 using the ClusterProfiler R package.

### Quantitative PCR evaluation and RNA isolation

Total RNA was isolated from CD4^+^ T cells using Trizol, and cDNA was synthesized using the RevertAid First-Strand cDNA Synthesis Kit (Thermo Fisher, K1622). Real-time PCR was performed using Fast SYBR Green Master Mix (Thermo Fisher, K0253), and gene expression levels were calculated using the 2^-^^ΔΔCt^ method. The primer sequences are listed in Table S4.

### Transmission electron microscopy

CD4^+^ T cells were fixed, washed, embedded in resin, sectioned and stained for transmission electron microscopy. Images were then digitally captured for analysis [17].

### CFSE staining

Cells were stained with CFSE (ThermoFisher, Cat No. C34554), washed with RPMI 1640 medium, and incubated with 2 × CFSE at 37 °C for 10 min. They were cultured for 96 h at 37 °C with or without PHA-P (30 µg/mL) and analyzed by flow cytometry. The proliferation index was calculated as: CFSE = (F1 − F2)/F1, where F1 is the fluorescence intensity before division and F2 is the intensity after division [18].

### Measurement of intracellular MDA

CD4^+^ T cells were separated from PBMCs of HDs and ATAAD patients by MACS. The cell lysates were measured with MDA detection kit (ThermoFisher, catalog no. EEA015) according to the manufacturer’s instructions.

### Iron ion detection

CD4^+^ T cells isolated from PBMCs were analyzed for iron ion detection using the ferrous ion content assay kit (Abcam, catalog no. ab83366) according to the manufacturer’s instructions. A wavelength of 520 nm was used to measure absorbance using spectrophotometry.

### Statistics

Sample sizes were determined on the basis of previous experiments using similar methodologies [9] and are detailed in each figure legend. Data are presented as the mean ± SEM for normally distributed continuous variables and *n* (%) for categorical variables. When comparing two groups, Student’s *t*-test was used for data comparisons. For paired data analysis, a paired *t*-test was performed. For comparisons of more than two groups, the ordinary one-way analysis of variance (ANOVA) test was utilized, and post-ANOVA pair-wise two-group comparisons were made using Tukey’s method. In order to investigate the relationship between CD4^+^ T cell subsets and clinical outcomes for AAD patients, Spearman’s rank correlation analysis was performed. All statistical analyzes were carried out using GraphPad Prism ver. 8. The level of statistical significance was set as: *, *P* < 0.05; **, *P* < 0.01; ***, *P* < 0.001; ****, *P* < 0.0001.

## Results

### Patient characteristics

One hundred and ten individuals, including patients with HDs (*n* = 55) and patients with ATAAD (*n* = 55), were enrolled in the study; their clinical characteristics are listed in Table S1. Analysis revealed that, except for a history of hypertension, there were no significant differences between ATAAD patients and healthy individuals in terms of age, gender, body mass index (BMI), coronary heart disease, diabetes, Marfan syndrome and smoking habits. These findings established a balanced baseline for further investigations into ATAAD-specific biomarkers and risk factors.

### CD4^+^ T cells from ATAAD patients are functionally impaired

**Fig. 1:**
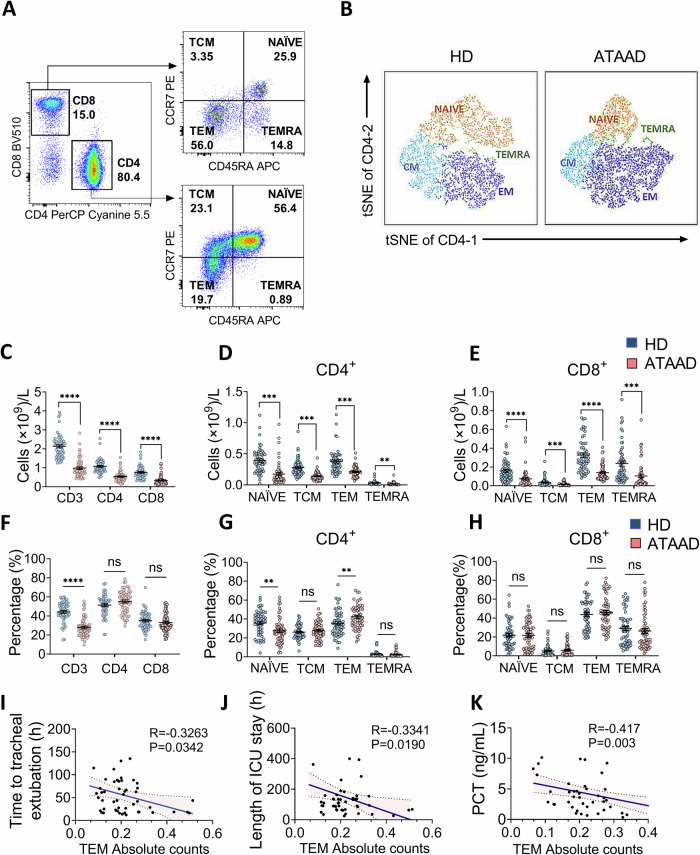
Abnormally differentiated CD4^+^ T cell subsets were correlated to clinical outcomes in ATAAD patients. **A** Gating strategy for the identification of CD4^+^ and CD8^+^ subpopulations by flow cytometry. The classification was carried out hierarchically: CD45RA^+^CCR7^+^ (NAÏVE), CD45RA^-^CCR7^+^ (TCM, central memory), CD45RA^-^CCR7^-^(TEM, effector memory) and CD45RA^+^CCR7^-^ (TEMRA, terminally differentiated effector memory). **B** t-Distributed Stochastic Neighbor Embedding (tSNE) plot of flow cytometry data showing CD4^+^ T cell phenotype distribution in ATAAD patients and HD. **C**–**E** Absolute counts of subgroups of total T cells (**C**), CD4^+^ T cells subpopulations (**D**), and CD8^+^ T cells subpopulations (**E**) in PBMCs from HDs and ATAAD patients (*n* = 55). **F**–**H** Percentage comparisons of subgroups of T cells (**F**), CD4^+^ T cells subpopulations (**G**), and CD8^+^ T cells subpopulations (**H**) in PBMCs from HDs and ATAAD patients (*n* = 55). **I**–**K** Correlation analyzes link absolute counts of CD4^+^ TEM cells to postoperative time to tracheal extubation (**I**), ICU stay length (**J**), and serum PCT concentration one day post-surgery (**K**) in ATAAD patients (*n* = 49). Each symbol represents one individual. Data are presented as mean ± SEM. Unpaired Student’s *t*-tests (C-H). **P < 0.01; ***P < 0.001; ****P < 0.0001. ICU intensive care unit, TCM central memory T cells, TEM effector memory T cells, TEMRA differentiated effector memory T cells.

Using the markers CD45RA and CCR7, changes in the functional populations of CD4^+^ and CD8^+^ T cells were assessed, including NAÏVE T cells (CD45RA^+^ CCR7^+^), central memory T cells (TCM, CD45RA^-^ CCR7^+^), effector memory T cells (TEM, CD45RA^-^ CCR7^-^), and differentiated effector memory T cells (TEMRA, CD45RA^+^ CCR7^-^) (Fig. 1A). These evaluations were further analyzed using t-distributed stochastic neighbor embedding (tSNE) maps (Fig. 1B). The absolute total counts of CD3^+^, CD4^+^ and CD8^+^ T lymphocyte in ATAAD patients were lower than for age- and gender-matched HDs (Fig. 1C). Moreover, the numbers of all CD4^+^ and CD8^+^ T cell subsets decreased (Fig. 1D and E). However, ATAAD patients exhibited a lower percentage of CD3^+^, NAÏVE CD4^+^ T cells but a higher percentage of TEM subsets as compared to HDs (Fig. 1F and G). In contrast, the percentage of CD8^+^ T lymphocyte subsets were comparable between ATAAD patients and HDs (Fig. 1H).

Correlation analyzes revealed negative associations between the CD4^+^ TEM cell number at admission and the time to extubation or ICU stay length following surgery (Fig. 1I and J). In addition, the number of TEM cells was negatively correlated to postoperative procalcitonin (PCT) levels (Fig. 1K). Thus, the abnormal differentiated CD4^+^ T cell subsets were implicated with perioperative infection and poor clinical outcomes.

**Fig. 2:**
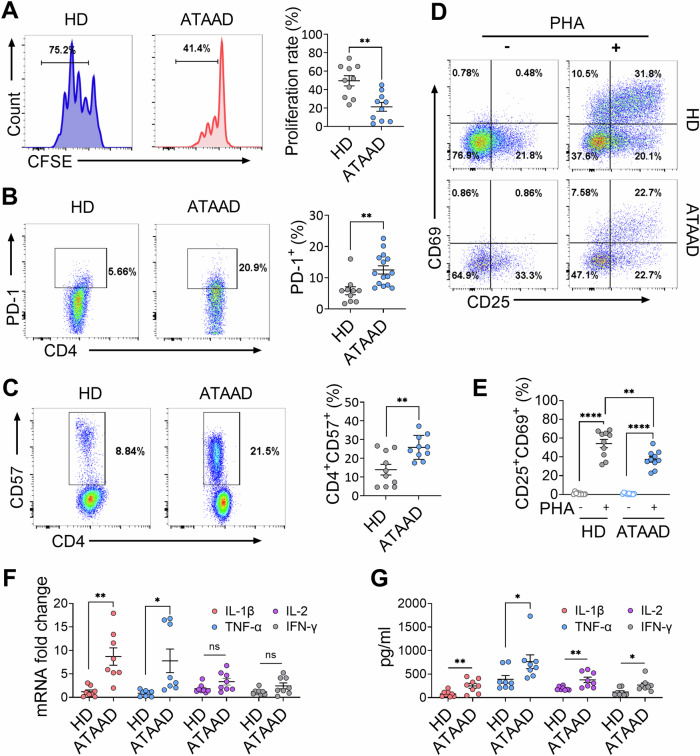
CD4^+^ T cells from ATAAD patients were functionally impaired. **A** The cell proliferation of CD4^+^ T cells was monitored after being cultured with PHA stimulation followed by addition of CFSE for 4 days. (HD, *n* = 10; ATAAD, *n* = 10). The proliferation rate was analyzed using FlowJoV10. **B**–**C** Flow cytometric quantification of PD-1 (**B**) (HD, *n* = 10; ATAAD, *n* = 10) and CD57 (**C**) (HD, *n* = 10; ATAAD, *n* = 10) expression in CD4^+^ T cells from HD and ATAAD patients. **D** CD4^+^ T cell activation was evaluated using flow cytometry to quantify CD25 and CD69 expressions 24 h after PHA treatment. **E** Quantification of activation percentage of CD4^+^ T cells (CD25^+^CD69^+^) (HD, *n* = 10; ATAAD, *n* = 10). **F**–**G** RT-PCR and ELISA assays were utilized to evaluate the expression levels of IL-1β, IL-2, TNF-α, and IFN-γ at the gene and protein levels in CD4^+^ T cells (HD, *n* = 8; ATAAD, *n* = 8). Data are given as the mean ± SEM. Unpaired Student’s *t*-tests (**A**–**C**, **F**–**G**) and one-way ANOVA (**E**) was performed. *P < 0.05; **P < 0.01; ***P < 0.001; ****P < 0.0001. ATAAD acute type-A aortic dissection, CFSE carboxyfluorescein succinimidyl ester, HD healthy donors, PD-1 programmed death-1, PHA phytohemagglutinin.

The functional profile of CD4^+^ T cells from ATAAD patients was evaluated by analyzing inhibitory checkpoint receptor expression, proliferation and activation capacity by using multiparametric flow cytometry. PHA was used to activate CD4^+^ T cells followed by carboxyfluorescein diacetate succinimidyl ester (CFSE) staining analysis. As shown, the proliferation rate of CD4^+^ T cells was greatly decreased after PHA treatment in ATAAD patients compared to HDs (Fig. 2A). The inhibitory markers PD-1 and CD57 [19] were found to be increased in CD4^+^ T cells from ATAAD patients compared to those from HDs (Fig. 2B and C). Notably, patients with lymphopenia demonstrated higher proportion of CD4^+^PD1^+^ T cells (Fig. S2). Among CD4^+^ T cells, the percentages of CD69^+^ and CD25^+^CD69^+^ T cells were lower in ATAAD patients in response to the stimulus, indicating impaired T cell activation (Fig. S3 and Fig. 2D–E).

Additionally, in CD4^+^ T cells from ATAAD patients, there was a significant increase in the mRNA expression of the proinflammatory cytokines IL-1β and TNF-α, whereas the mRNA expression levels of IL-2 and IFN-γ were comparable between ATAAD patients and HDs (Fig. 2F). ELISA analysis further demonstrated that the intracellular protein levels of these cytokines were elevated in CD4^+^ T cells (Fig. 2G). Overall, these results indicated that CD4^+^ T cells exhibit a proinflammatory state and intrinsic activation defect that limited their expansion in ATAAD patients.

### The occurrence of ferroptosis dampened CD4^+^ T cell function in ATAAD patients

**Fig. 3:**
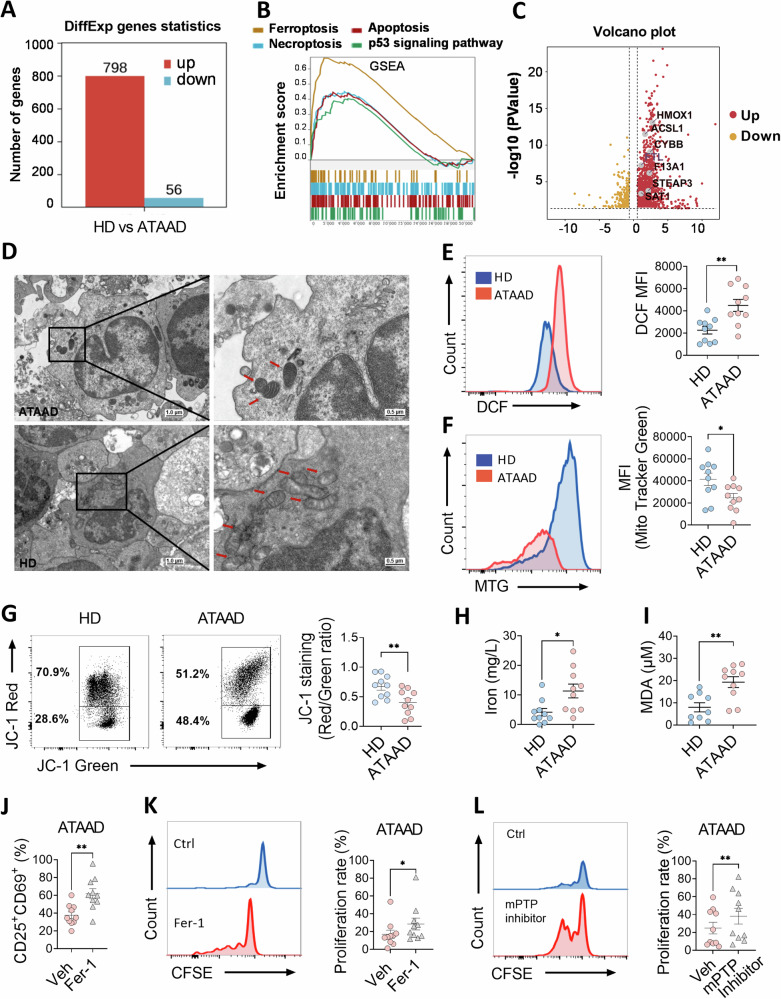
Impaired functions of CD4^+^ T cells in ATAAD patients were driven by ferroptosis. RNA-seq analysis of CD4^+^ T cells harvested from HDs and ATAAD patients (HD, *n* = 4; ATAAD, *n* = 6). **A** Quantification of DEGs in CD4^+^ T cells from HDs and ATAAD patients, categorized into upregulated and downregulated genes. **B** Enrichment plots of ferroptosis and other types of cell death, identified by the GSEA computational method. **C** Volcano plot showing the DEGs. Gray dots represent the genes upregulated in CD4^+^ T cells harvested from ATAAD patients. **D** Representative electron micrographs of CD4^+^ T cells in HDs (down) and ATAAD patients (up). The scale bar represents 1.0 μm (left) and 500 nm (right). **E** CD4^+^ T cells were analyzed for cytosolic cytoplasmic ROS (HD, *n* = 10; ATAAD, *n* = 10). **F** The mitochondrial mass of CD4^+^ T cells was examined using MitoTracker Green (HD, *n* = 10; ATAAD, *n* = 10). **G** Mitochondrial membrane potential in CD4^+^ T cells was assessed using JC-1 staining (HD, *n* = 10; ATAAD, *n* = 10). **H**–**I**. CD4^+^ T cells were analyzed for iron **H** (HD, *n* = 10; ATAAD, *n* = 10), lipid peroxidation **I** (HD, *n* = 10; ATAAD, *n* = 10). **J** Flow cytometric quantification was performed to assess the activation percentages of CD4^+^ T cells (CD25^+^CD69^+^) in vitro pretreated or not pretreated with Fer-1 (5 μM) (ATAAD, *n* = 10). **K** CD4^+^ T cell proliferation in ATAAD patients in the presence or absence of Fer-1 (ATAAD, *n* = 10). **L**. Proliferation of CD4^+^ T cells in ATAAD patients in the presence or not of mPTP opening inhibitor ER-000444793 (100 nM) (ATAAD, *n* = 10). Each symbol represents one individual. Data are presented as the mean ± SEM. Unpaired and paired Student’s *t*-tests were performed. *P < 0.05; **P < 0.01; ***P < 0.001; ****P < 0.0001. ATAAD acute type-A aortic dissection, DEGs differentially expressed genes, Fer-1 ferrostatin-1, HD healthy donors, MDA malondialdehyde, mPTP mitochondrial permeability transition pore, MTG mitotracker green, ROS reactive oxygen species.

To clarify the mechanisms involved in CD4^+^ T cell hypofunction, RNA-seq was performed on cells isolated from HDs and ATAAD patients. A total of 789 genes were upregulated and 54 genes downregulated among the 15,692 genes analyzed (Fig. 3A and Fig. S4A). GSEA assay showed that gene signatures of ferroptosis were enriched and more dramatically altered than for other cell death pathways, such as apoptosis and necrosis (Fig. 3B). Subsequently, ferroptotic DEGs were identified as being upregulated in the volcano plot and were subsequently used to construct a heatmap (Fig. 3C and Fig. S4B). Furthermore, in CD4^+^ T cells isolated from patients with ATAAD, ferroptotic genes such as *ACSL1*, *SATA1*, *STEAP3*, *CYBB* and *F13A1* were upregulated (Fig. S4C).

Mitochondrial dysfunction is a hallmark of ferroptosis [20]. Electron microscopy was used to examine the ultrastructure of mitochondria in CD4^+^ T cells. As demonstrated, a sizable fraction of CD4^+^ T cells in ATAAD patients had ferroptosis-specific morphological traits, such as the development of mitochondrial vacuoles with elevated mitochondrial membrane density and the elimination of mitochondrial cristae (Fig. 3D). Mitochondria are the primary organelles responsible for ROS production. Comparative analysis revealed significantly higher cytosolic ROS concentrations in CD4^**+**^ T cells from ATAAD patients than in HDs (Fig. 3E). RNA sequencing data further revealed mitochondrial dysfunction in CD4^**+**^ T cells of ATAAD patients, characterized by differential enrichment or expression of genes associated with mitochondrial function (Fig. S4D and E). Moreover, mitochondrial mass in ATAAD patients, assessed using MitoTracker Green, was found to be reduced compared to HDs (Fig. 3F), and this was accompanied by a compromised ΔΨm, as evidenced by a decrease in JC-1 staining fluorescence (Fig. 3G). Finally, elevated levels of intracellular iron (Fig. 3H) and MDA (Fig. 3I), markers of lipid peroxidation in CD4^**+**^ T cells from ATAAD patients, additionally indicated the occurrence of ferroptosis.

To elucidate further the impact of ferroptosis on the functionality of CD4^**+**^ T cells, the effect of inhibiting ferroptosis on the rejuvenation of hyporesponsive CD4^**+**^ T cells was evaluated. As demonstrated, the ferroptosis inhibitor, Fer-1, increased the percentage of CD69^+^ and CD25^**+**^CD69^**+**^ T cells in ATAAD patients following PHA treatment (Fig. S5 and Fig. 3J). Additionally, Fer-1 reversed the low proliferation rate of CD4^**+**^ T cells in AAD patients, as indicated by CFSE analysis (Fig. 3K). Consistently, the mPTP opening inhibitor, ER-000444793, also alleviated the impaired proliferation of CD4^**+**^ T cells in ATAAD patients (Fig. 3L). Overall, these results strongly suggest that ferroptosis impairs the function of CD4^**+**^ T cells.

### FAs trigger ferroptosis and hypofunction in CD4+ T cells from ATAAD patients

**Fig. 4:**
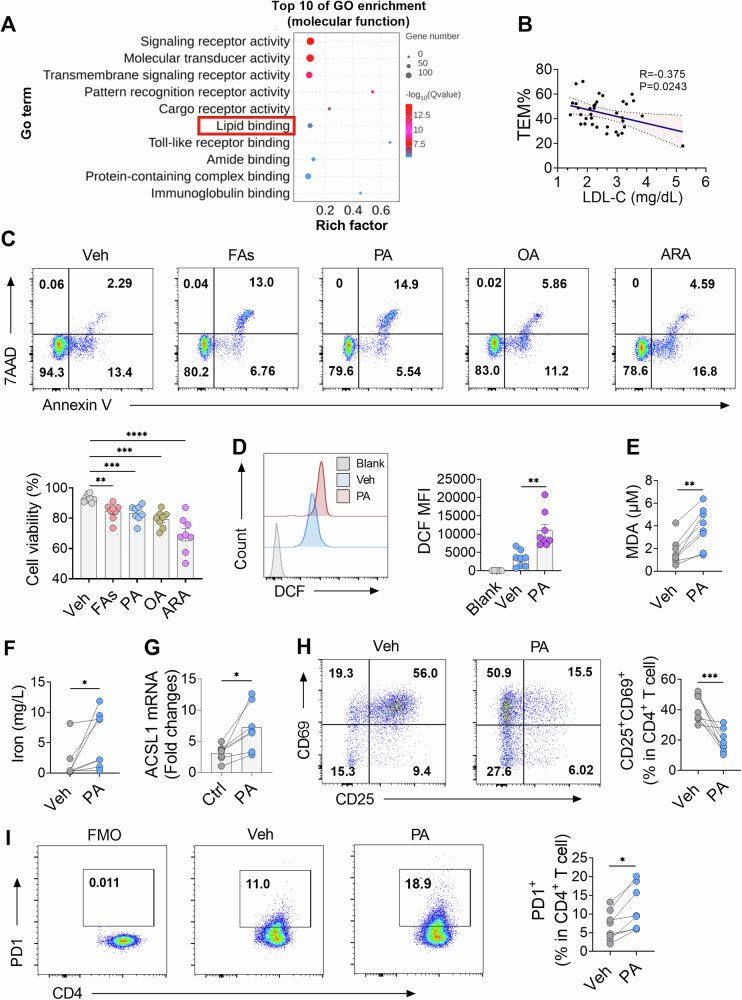
Palmitic acid-induced ferroptosis and hypofunction of CD4^+^ T cells in ATAAD patients. **A** The top 10 GO pathways analysis showed enrichment of DEGs in lipid binding. **B** Correlation analysis of the percentage of CD4^+^ TEM cells with LDL-c (*n* = 36). **C** Flow cytometric quantification of cell viability in CD4^+^ T cells from ATAAD (Annexin V^-^ 7AAD^-^, live cells), treated with FAs (50 μM), PA (50 μM), OA (50 μM) and ARA (50 μM) for 24 h (*n* = 8). **D** Flow cytometric quantification of ROS in ATAAD CD4^+^ T cells treated with PA and FAs (*n* = 8). **E**–**F**. Assessment of PA effects on MDA concentration **E** and iron levels **F** in CD4^+^ T cells from ATAAD patients (*n* = 8). **G** ACSL1 mRNA in CD4^+^ T cells isolated from HD and ATAAD patients measured by RT-PCR (*n* = 8). **H** Flow cytometric quantification was performed to assess the activation percentages of CD4^+^ T cells (CD25^+^CD69^+^) after stimulation with PA (*n* = 8). **I** Effects of PA on PD-1 expression in CD4^+^ T cells isolated from ATAAD patients (*n* = 8). Data reflect 3 independent measurements. Each symbol represents one individual. Data are presented as the mean ± SEM. One-way ANOVA (**C**) and paired Student’s *t*-test (**D**–**I**) were performed. *P < 0.05; **P < 0.01; ***P < 0.001; ****P < 0.0001. FAs were a mixture of PA, OA, and ARA in equal concentrations of 50 μM. ATAAD acute type-A aortic dissection, ARA arachidonic acid, OA oleic acid, PA palmitic acid, DCF dichlorofluorescein, FAs fatty acids, GO gene ontology, LDL-c low-density lipoprotein cholesterol, MFI mean fluorescence intensity, Veh vehicle, 7AAD 7-aminoactinomycin D.

Hyperlipidemia is recognized as a prevalent risk factor for aortic dissection [21, 22]. Analysis of published transcriptomic data (GSE190635) from aortic tissues of AAD patients and HDs highlighted that the top 20 enriched pathways included ether lipid metabolism, fat digestion and absorption and regulation of lipolysis in adipocytes (Fig. S6). Additionally, evidence indicating that bariatric surgery leads to fewer hospital admissions for ATAAD patients supports the link between hyperglycemia and aortic dissection [23]. Consequently, the impact of hyperlipidemia on CD4^+^ T cell abnormalities was investigated. RNA-seq analysis indicated that DEGs in CD4^+^ T cells were enriched in lipid binding and lipid metabolism-related GO terms (Fig. 4A and Fig. S7). A negative correlation was also observed between the percentage of TEM cells and levels of low-density lipoprotein cholesterol (LDL-c) in ATAAD patients (Fig. 4B), suggesting a significant role of lipid metabolism in the abnormalities of CD4^+^ T cells obtained from ATAAD patients.

Overconsumption of fats, particularly saturated FAs such as PA, has been linked to lipotoxicity-related conditions including diabetes and cardiovascular diseases [24, 25]. The effects of FA mixtures, PA, OA and ARA on the viability of CD4^+^ T cells were investigated. The study specifically measured the percentage of living cells (Annexin V^-^7AAD^-^). As expected, treatment with FA mixture, PA, OA and ARA reduced CD4^+^ T cell viability (Fig. 4C). To clarify the specific mechanism of compromised cell viability induced by PA, additional experiments focused on its impact on CD4^+^ T cells. As expected, treatment with PA significantly increased ROS production (Fig. 4D), MDA and iron levels in these cells (Fig. 4E–F). Moreover, the gene expression of ACSL1, a ciritical molecular mediating ferroptosis through the regulation of lipid metabolism [26], was elevated in response to PA (Fig. 4G), further indicating that PA promotes ferroptosis in CD4^+^ T cells.

Given the impact of FAs on T cell ferroptosis in AAD patients, it was hypothesized that FAs could lead to CD4^+^ T cell dysfunction. Supporting this hypothesis, PA treatment was found to inhibit T cell activation, as evidenced by a decreased proportion of CD25^+^, CD69^+^ and CD25^+^CD69^+^ cells following PHA stimulation (Fig. S8 and Fig. 4H). Additionally, PA treatment increased the proportion of PD1^+^CD4^+^ T cells in AAD patients (Fig. 4I). These results indicated that elevated FA levels induced ferroptosis and contributed to the hypofunctional phenotype of CD4^+^ T cells in ATAAD patients.

### Inhibition of ferroptosis attenuated CD4^+^ T cells hypofunction induced by PA

**Fig. 5:**
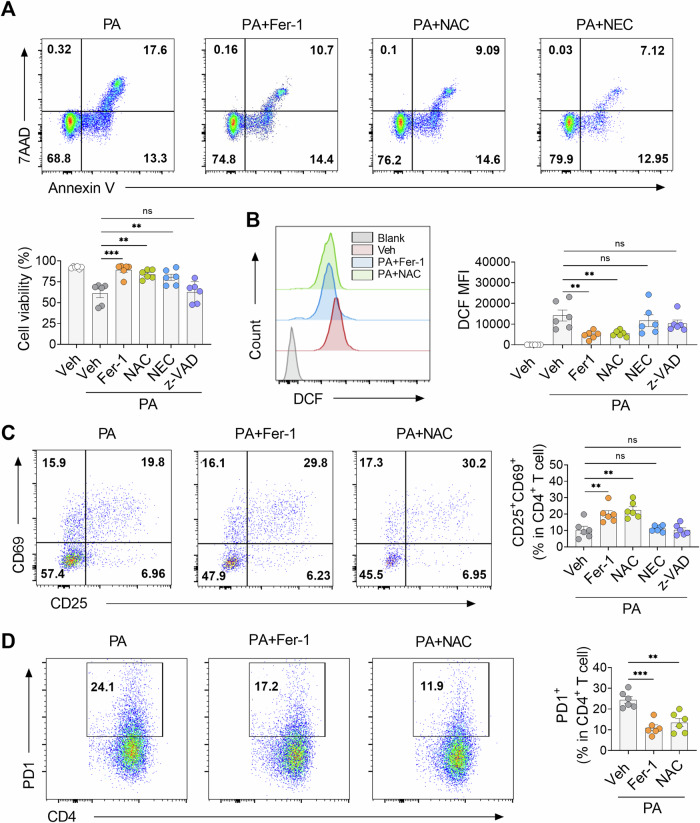
CD4^+^ T cell hypofunction induced by PA could be reversed by N-acetylcysteine and ferrostatin-1. **A** Flow cytometric analysis of CD4^+^ T cell viability after 4-h pre-treatment with ferrostatin-1 (5 µM), z-VAD-fmk (10 µM), NAC (30 mM), or NEC (20 µM) followed by PA exposure (*n* = 6). **B** Flow cytometric quantification of ROS in ATAAD CD4^+^ T cells with or without addition of indicated reagent (*n* = 6). **C** Flow cytometric quantification of early activation of CD4^+^ T cells in ATAAD patients with or without addition of indicated reagent (*n* = 6). **D** Flow cytometric quantification of PD-1 expression in CD4^+^ T cells from ATAAD patients with or without addition of indicated reagent (*n* = 6). Each symbol represents one individual. Data reflect 3 independent measurements and presented as the mean ± SEM. One-way ANOVA was performed. *P < 0.05; **P < 0.01; ***P < 0.001; ****P < 0.0001. ATAAD acute type-A aortic dissection, MFI mean fluorescence intensity, NAC n-acetylcysteine, NEC necrostatin, Z-VAD-fmk benzyloxycarbonyl-Val-Ala-Asp(OMe)-fluoromethylketone.

To explore the impact of blocking ferroptosis on PA-induced CD4^+^ T cell dysfunction, inhibitors for ferroptosis, antioxidants, necrosis and apoptosis were utilized. The results showed that the ferroptosis inhibitor Fer-1, the antioxidants NAC, and necrosis inhibitor NEC restored the compromised viability of CD4^+^ T cells caused by PA (Fig. 5A). Interestingly, Fer-1 and NAC, rather than NEC, were shown to significantly reduce ROS generation in CD4^+^ T cells (Fig. 5B). Additionally, treatment with Fer-1 and NAC also restored PA-suppressed CD4^+^ T cell activation, as evidenced by an increased proportion of the CD25^+^, CD69^+^ and CD25^+^CD69^+^ subpopulation (Fig. S9 and Fig. 5C), and inhibited their PD-1 expression (Fig. 5D). In contrast, neither the apoptosis inhibitor Z-VAD nor the necrosis inhibitor NEC affected the activation of CD4^+^ T cells (Fig. 5C). Overall, these findings indicate that PA induced ferroptosis plays a central role in mediating T cell dysfunction.

### FAs promoted CD4^+^ T cell ferroptosis through CD36 in ATAAD patients

**Fig. 6:**
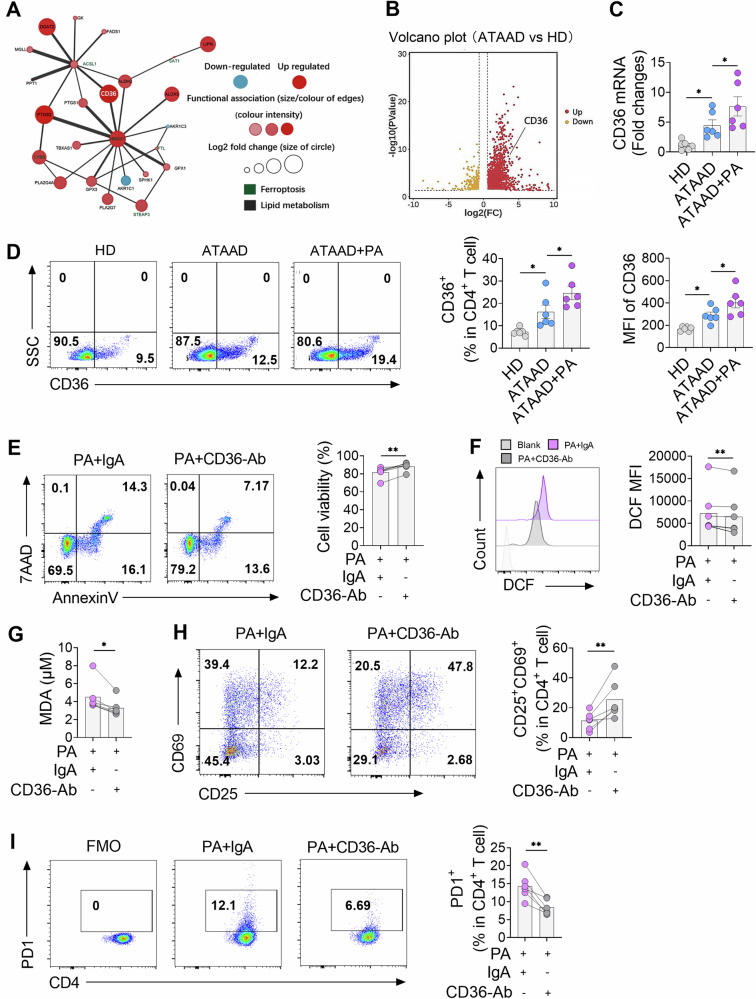
CD36-mediated palmitic acid-induced ferroptosis and hypofunction of CD4^+^ T cells. **A** RNA sequencing and PPI analysis revealed pathways involved in ferroptosis and lipid metabolism in CD4^+^ T cells from ATAAD patients compared to HD. **B** Volcano plots show genes differentially expressed between groups, including CD36. **C**–**D** CD36 expression in CD4^+^ T cells, assessed by RT-PCR (**C**) and FCM (**D**), with and without PA treatment (HD, *n* = 6; ATAAD, *n* = 6 for RT-PCR; HD, *n* = 6; ATAAD, n = 6 for FCM). **E** Cell viability in CD4^+^ T cells treated with CD36-mAb and exposed to PA, analyzed via FCM (ATAAD, *n* = 6 for viability). **F**–**G** Impact of CD36 blockade on ROS generation (**F**) and MDA levels (**G**) in PA-induced CD4^+^ T cells (ATAAD, MDA: *n* = 6; ATAAD, ROS: *n* = 6). **H**–**I** Effects of CD36 inhibition on early T cell activation (**H**) and PD-1 induction (**I**), under PA influence (ATAAD, Activation: *n* = 6; PD-1: *n* = 6). Each symbol represents one individual. Data reflect at least 3 independent experiments and presented as the mean ± SEM. Statistical analysis by One-way ANOVA (**C**, **D**), paired Student’s *t*-test (**E**–**I**). *P < 0.05; **P < 0.01. ATAAD acute type-A aortic dissection, CD36-mAb CD36 monoclonal antibody, FCM flow cytometry, HD healthy donor, PA palmitic acid.

To investigate the mechanism of FA-mediated T cell ferroptosis, protein-protein interaction (PPI) analysis alongside RNA sequencing revealed that CD36 is a crucial gene linking lipid binding and ferroptosis (Fig. 6A). CD36, a key receptor involved in FA uptake and widely expressed in various immune cells, is recognized as a critical molecular target driving ferroptosis [27]. Analysis of DEGs and RT-PCR further revealed significant upregulation of CD36 in ATAAD patients (Fig. 6B and C). Notably, patients with lymphopenia demonstrated higher CD36 expression (Fig. S10). Treatment with PA further increased both the expression and percentage of CD36 in CD4^+^ T cells of ATAAD patients (Fig. 6D).

To investigate further whether CD36 was involved in PA-induced CD4^+^ T cell ferroptosis in ATAAD, CD36 blockade by a mAb alleviated ferroptosis, restoring cell viability (Fig. 6E), and decreased ROS production and the MDA concentration following PA treatment (Fig. 6F,G). Additionally, blocking CD36 enhanced T cell activation and downregulated PD-1 expression compared to the IgA control (Fig. S11 and Fig. 6H,I). These findings collectively suggest that CD36 plays a pivotal role in FA-mediated ferroptosis and T cell hypofunction in ATAAD patients.

## Discussion

In AAD, disruption of the media and an intimal tear of the aorta produce an intense inflammatory response [28]. Sterile inflammation caused by aortic rupture produces enormous amounts of damage-related molecular patterns (such as in mitochondrial DNA), which impairs the immune system’s ability to respond [6, 29]. Given the unpredictable nature and difficulty in preventing ATAAD, identifying factors to enhance the postoperative outcomes of ATAAD patients holds great clinical significance. In the present study, we demonstrated that ATAAD patients experience disruption of homeostasis in CD4^+^ T cells, characterized by hypofunction and an inflammatory state. This manifests as dampened activity and proliferation capacity, elevated expression of inhibitory checkpoint receptor and aberrant T cell differentiation that correlated with adverse outcomes for ATAAD patients. Moreover, CD4^+^ T cell hypofunction in ATAAD patients is driven by ferroptosis. Mechanistically, upregulated CD36 in ATAAD patients dramatically sensitizes these cells to ferroptosis through the uptake of palmitate. Thus, our results demonstrated that CD36-mediated ferroptosis contributes to the hypofunction of CD4^+^ T cells, which may be associated with poor postoperative outcomes.

After exposure to antigen, NAÏVE cells become activated and differentiate into effector cells, and ultimately memory T cells. TEM cells migrate to and reside in target organs such as the lung, where they provide rapid effector functions. Their balance is crucial for orchestrating immune responses and the subsequent tissue damage in various inflammatory diseases, such as autoimmunity, sepsis and trauma. The clinical implications of CD4^+^ T cell activation diversification during differentiation in ATAAD patients are unclear, despite evidence linking T cell dysregulation and polarization to the occurrence of AAD [8, 30]. In the present study, ATAAD patients had a higher percentage of NAÏVE CD4^+^ T cells and lower frequency of CD4^+^ TEM cells, suggesting dampened effector functions. We also found a negative correlation between CD4^+^ TEM cell counts at admission and the duration of ICU stay, time to extubation, and infection marker PCT. These clinical correlations suggest that an imbalance in CD4^+^ T cell deficiency and differentiation may increase susceptibility to postoperative secondary infection and worsen prognosis in ATAAD patients. Several processes might explain the altered proportions of CD4^+^ subsets observed, including cell redistribution, decreased proliferation and reduced survival of TEM cells. We also identified intrinsic activation and proliferation defects in CD4^+^ T cells, potentially limiting their expansion and differentiation in ATAAD patients. Further study is needed to determine the mechanism behind this altered homeostasis and its effects on host responses to potential organ injury.

Although our previous study found that lymphopenia is common and associated with poor outcomes in ATAAD patients [9], the functional state of T cells remains largely unknown. In this study, CD4^+^ T cells in ATAAD patients exhibited a defect that compromised homeostasis and function, impaired proliferation and activation, aberrant differentiation and high inhibitory receptor expression. These features resemble T cell exhaustion, a state of functional hyporesponsiveness. The cells in this state progressively lose effector functions and self-renewal capacity, thereby inhibiting the immune response and facilitating infection persistence or poor tumor control [31–35]. However, unlike typical impaired cytokine production in exhausted T cells, CD4^+^ T cells from ATAAD patients showed increased expression of cytokines, notably proinflammatory cytokines. This difference may be because these CD4^+^ T cell alterations caused by acute aortic injury are in an early stage of exhaustion progression, akin to the gradual functional decline of CD4^+^ T cells over time post-infection. For instance, early stages of CD4^+^ T cell exhaustion following *Schistosoma japonicum* infection showed increased IL-6 and IFN-γ expression, accompanied by a gradual increase in PD-1 expression. In contrast, during the later stages, most cytokines were suppressed in exhausted T cells [36]. Our results highlight that an exhaustion-like state may also occur in acute states, such as acute aortic injury. We speculate that defects in CD4^+^ T cell activity, proliferation and differentiation may increase susceptibility to and facilitate secondary infections, while the inflammatory state of CD4^+^ T cells could worsen organ damage. Both factors may intertwine to contribute to the poor outcomes of ATAAD patients.

Ferroptosis is iron-dependent and caused by lipid peroxidation-mediated damage of the cell membrane [11]. The effects of ferroptosis on T cell function seem to be context-dependent. Ferroptosis can enhance T cell activation in multiple sclerosis, yet it has also been found to impair CD8^+^ T cell effector functions and anti-tumor capabilities [27, 37]. However, the involvement of ferroptosis in T cell abnormalities within ATAAD remains to be elucidated. Mitochondrial damage and lipid peroxidation are the two most prominent features of ferroptosis. The present study observed shrunken and damaged mitochondria, disruptions in mitochondrial membrane potential and reduced mitochondrial numbers, indicating ferroptosis and mitochondrial damage. Conversely, inhibiting ferroptosis or the mPTP opening inhibitor enhanced the impaired proliferation and activation of CD4^+^ T cells. These results suggest that mitochondrial dysfunction-mediated ferroptosis may contribute to CD4^+^ T cell fatigue in ATAAD patients.

Ferroptosis exacerbates ATAAD progression through multiple pathways. Ferroptosis is associated with inflammatory signaling activation, including NF-κB, inflammasome, and cGAS-STING [38], which may contribute to damage of the aortic wall. Additionally, CD4^+^ T cells are critical for tissue repair after injury [38]. Dysfunction and loss of CD4^+^ T cells induced by ferroptosis may hinder the repair process and increase the risk of complications. Furthermore, in addition to pathogenic ferroptosis in circulating CD4^+^ T cells, ferroptosis in aortic dissection lesions, particularly within smooth muscle cells, is also noteworthy [39–41]. Ferroptosis inhibitors have also shown promise for the treatment of AAD in various contexts [42–45].

Obesity and dyslipidemia are the major risk factors for the progression of aortic dissection and aneurysm [46–48]. Our study further implicates CD36 involvement in driving the process of dysfunction and ferroptosis induced by FAs. CD36 is a scavenger receptor that acts as a transporter of FAs and oxidized lipids, and plays a vital role in atherosclerosis and metabolic disorders [49–52]. Blocking CD36 in CD8^+^ T cells effectively restored their anti-tumor activity without influencing PD-1 expression in tumor-infiltrating CD8^+^ lymphocytes [49]. In contrast, our study has shown that blocking the increased CD36 using an antibody restored the activity of CD4^+^ T cell and downregulated PD-1 expression. The inconsistency in the results may be attributable to several factors related to cell type specificity, differences in the microenvironment, and the model systems used (e.g., human vs. animal models). Building upon a recent study that unveiled the pivotal role of CD36 in regulating ferroptosis among tumor-infiltrating CD8^+^ T cells [27], the present study further identified the critical function of CD36 in mediating ferroptosis in CD4^+^ T cells, specifically within the context of ATAAD. Our data not only expanded the scope of CD36’s influence across different T cell subsets, but also shed light on how FAs contribute to T cell dysfunction and adverse outcomes in ATAAD.

Our study had certain limitations. Due to the lymphopenic state of the patients, the number of CD4^+^ T cells obtained from blood was low, which hindered the further elucidation of the detailed underlying mechanisms and examination of the susceptibility of ferroptosis in different subsets such as TEM CD4^+^ T subsets. Additionally, the characteristics and intrinsic mechanisms of CD4^+^ T cells observed in this study represent adaptive responses in ATAAD, and it remains to be determined whether these findings can be generalized to other types of aortic dissection. Furthermore, the lack of predictive risk factors for ATAAD and the reliance on surgical treatment [53] underscore the need to explore molecular mechanisms to devise new postoperative strategies. However, the lack of suitable animal models to mimic perioperative pathological scenarios in ATAAD patients limits the clinical applicability of our findings. Therefore, constructing ideal ATAAD surgery animal models will be crucial for future investigations into T cell dysfunction and outcomes.

## Conclusion

**Fig. 7:**
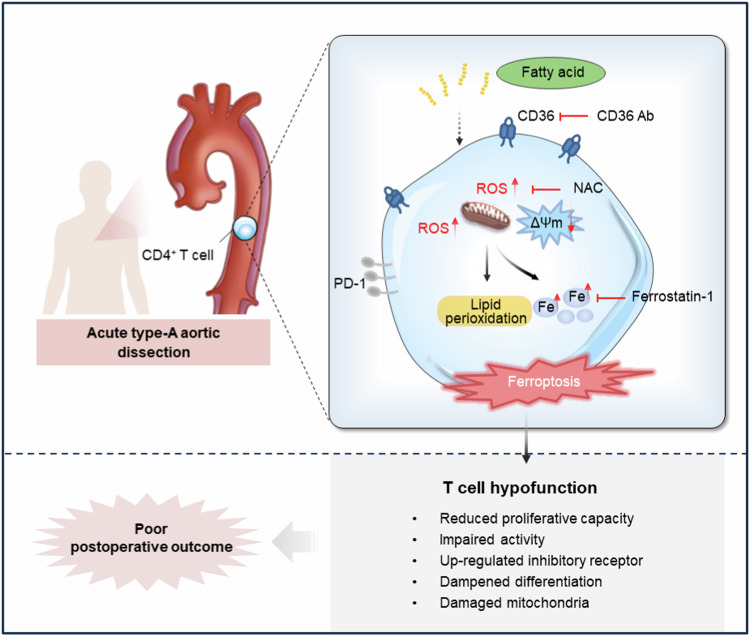
Schematic representation elucidates a vital role of ferroptosis in CD4^+^ T cell hypofunction, a novel immunological feature in ATAAD. Increased CD36 expression sensitizes CD4^+^ T cells to ferroptosis by uptake of fatty acids, which drives CD4^+^ T cell hypofunction that is associated with poor postoperative outcomes in ATAAD patients. Targeting the CD36-ferroptosis pathway is a potential anti-T cell hypofunction approach. ATAAD acute type-A aortic dissection, CD36 Ab CD36 antibody, NAC n-acetylcysteine, PD-1 programmed cell death protein 1, ROS reactive oxygen species, ΔΨm mitochondrial membrane potential.

In summary, the study highlighted the adaptive immune response in CD4^+^ T cells following acute artery injury, characterized by hypofunction and inflammatory responses. Elevated CD36 expression by FAs drives this impaired functionality, increasing the susceptibility of CD4^+^ T cells to ferroptosis. Thus, the results suggest that targeting CD36 and ferroptosis to enhance CD4^+^ T cell function may represent a promising therapeutic strategy to improve postoperative outcomes in ATAAD patients (Fig. 7). Additionally, our findings underscore the importance of managing dyslipidemia to enhance clinical outcomes for these patients.

## Supplementary information

Supplementary Figures and Legends

Supplementary Tables and Legends

## Data Availability

The datasets generated and/or analyzed during the current study are available from the corresponding authors on reasonable request.
