# Supplementary material for: CD36-mediated ferroptosis destabilizes CD4+ T cell homeostasis in acute Stanford type-A aortic dissection
Source: Cell Death Dis. 2024 Sep 12;15(9):669. doi: 10.1038/s41419-024-07022-9 (PMC11392947; doi:10.1038/s41419-024-07022-9)
Supplement: Supplementary file 1 — Supplementary Figures and Legends [file 41419_2024_7022_MOESM1_ESM.docx]

**Supplemental Figures**


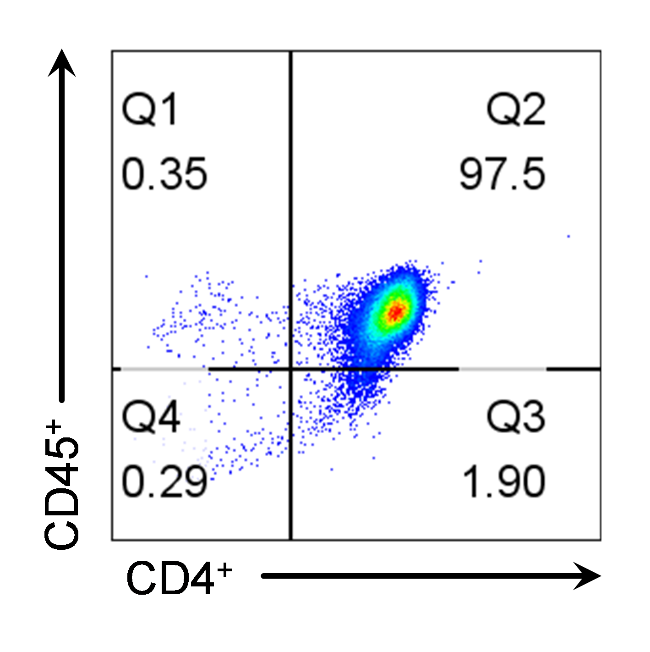


**Fig. S1. Purity of magnetically separated CD4^+^ T cells**

Flow cytometry analysis of CD4^+^ T cells isolated from PBMCs using magnetic bead separation.


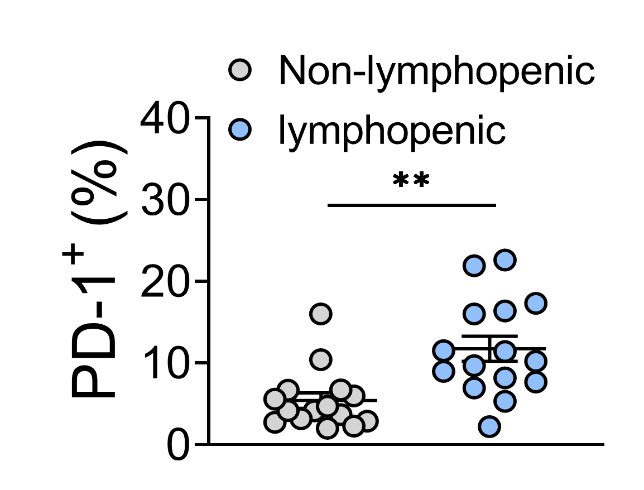


**Fig. S2. PD-1 expression on CD4+ T cells from ATAAD patients with or without lymphopenia**PD-1 expression was evaluated by flow cytometry in CD4+ T cells from ATAAD patients, grouped as non-lymphopenic (n=15) and lymphopenic (n=15). Unpaired student’s t-tests was performed.**P < 0.01. PD-1, programmed death-1.


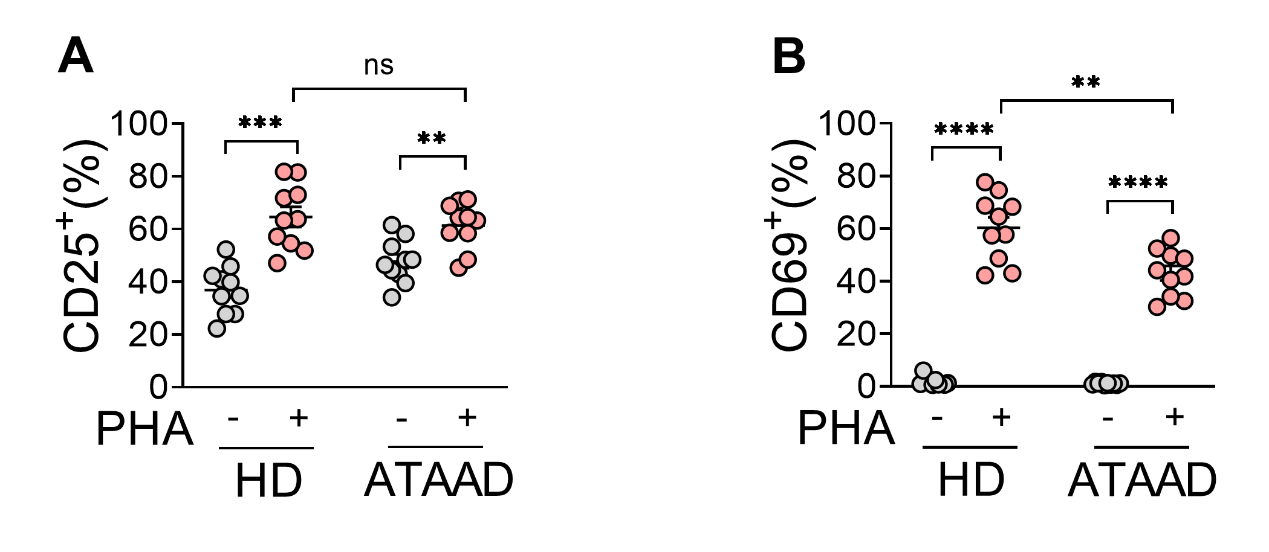


**Fig. S3. Comparison of CD25 and CD69 expression on CD4+ T cells between HDs and ATAAD patients**The proportion of CD4+ T cells expressing CD25+ and CD69+ was evaluated by flow cytometry in samples from HDs (n=10) and patients with ATAAD (n=10). One-way ANOVA was performed. *P<0.05; **P<0.01; ***P < 0.001; ****P<0.0001. ATAAD, type A acute aortic dissection; HD, healthy donors; PHA-P, phytohemagglutinin-P.


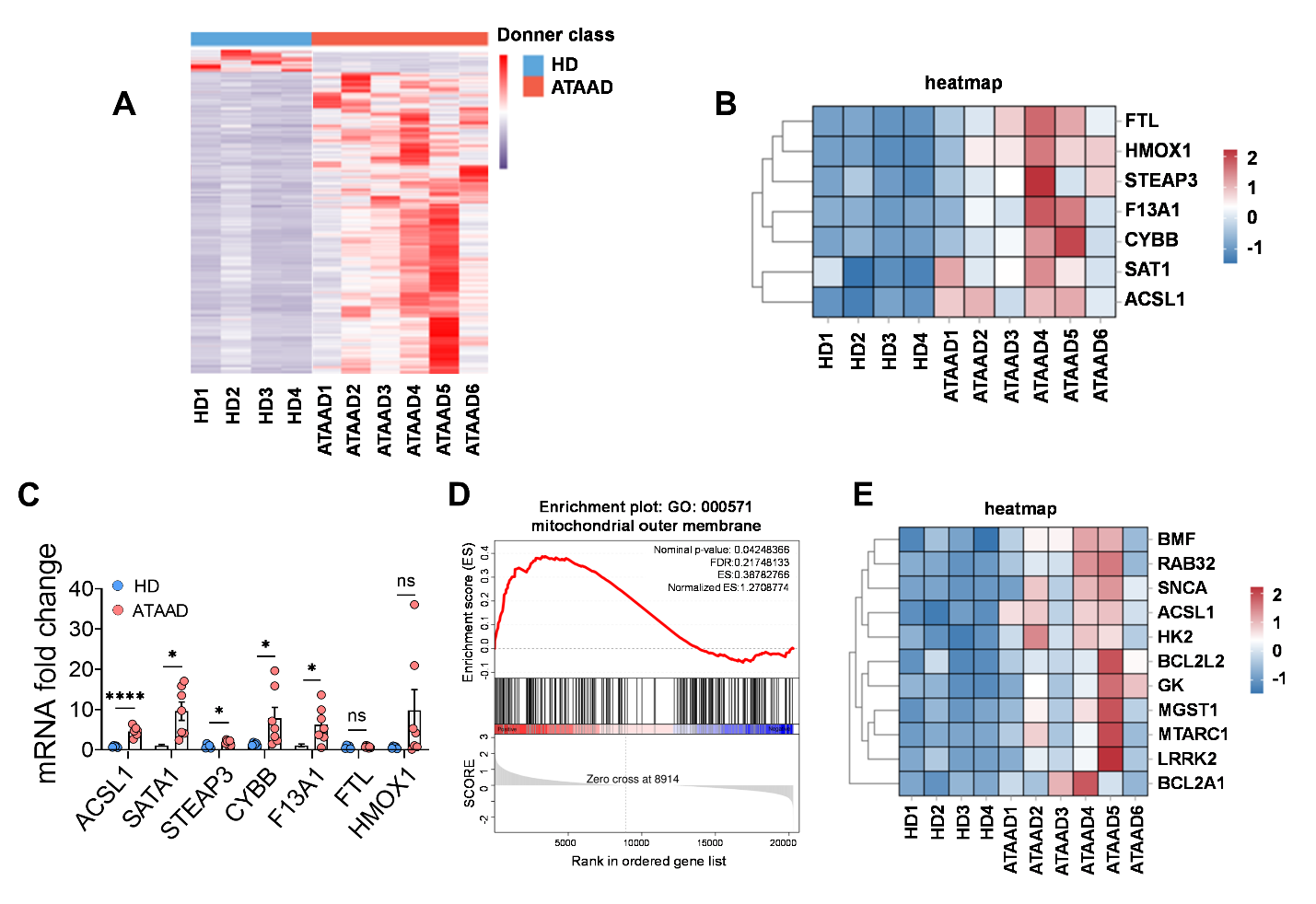


**Fig. S4. RNA sequencing analysis of ferroptosis and mitochondrial differential genes in CD4+ T cells from HDs and ATAAD patients**CD4+ T Cells from HDs and ATAAD patients were used for RNAseq analysis. A. Heatmap illustrating DEGs between HD and ATAAD patients, displaying patterns of upregulation and downregulation. B-C. Heatmap and RT-PCR (HD, n=5；ATAAD, n=7) showing expression levels of ferroptosis-associated genes. D. GSEA of the mitochondrial outer membrane-related gene sets. E. Heatmap presenting profiles of mitochondrial damage-related genes. Unpaired student’s t-tests were performed. *P<0.05; **P<0.01; ***P < 0.001; ****P<0.0001. ATAAD, type A acute aortic dissection; HD, healthy donors.


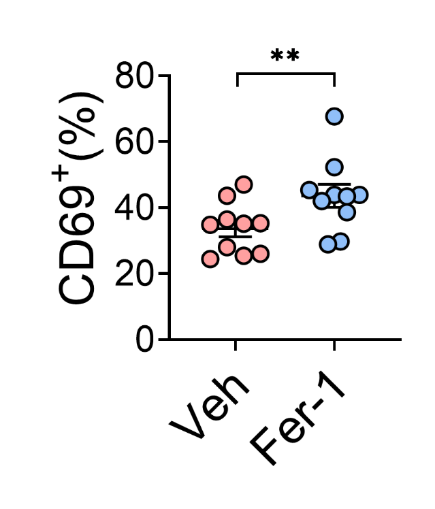


**Fig. S5. Restoration of CD4+ T cell activity by ferrostatin-1 in ATAAD patients**

Co-cultured for 24 hours under PHA, with or without Fer-1. CD4+ T cell activation was evaluated by analyzing the proportion of CD69+ cells (ATAAD, n=10). Paired student’s t-tests was performed. *P<0.05; **P<0.01. Fer-1, ferrostatin-1.


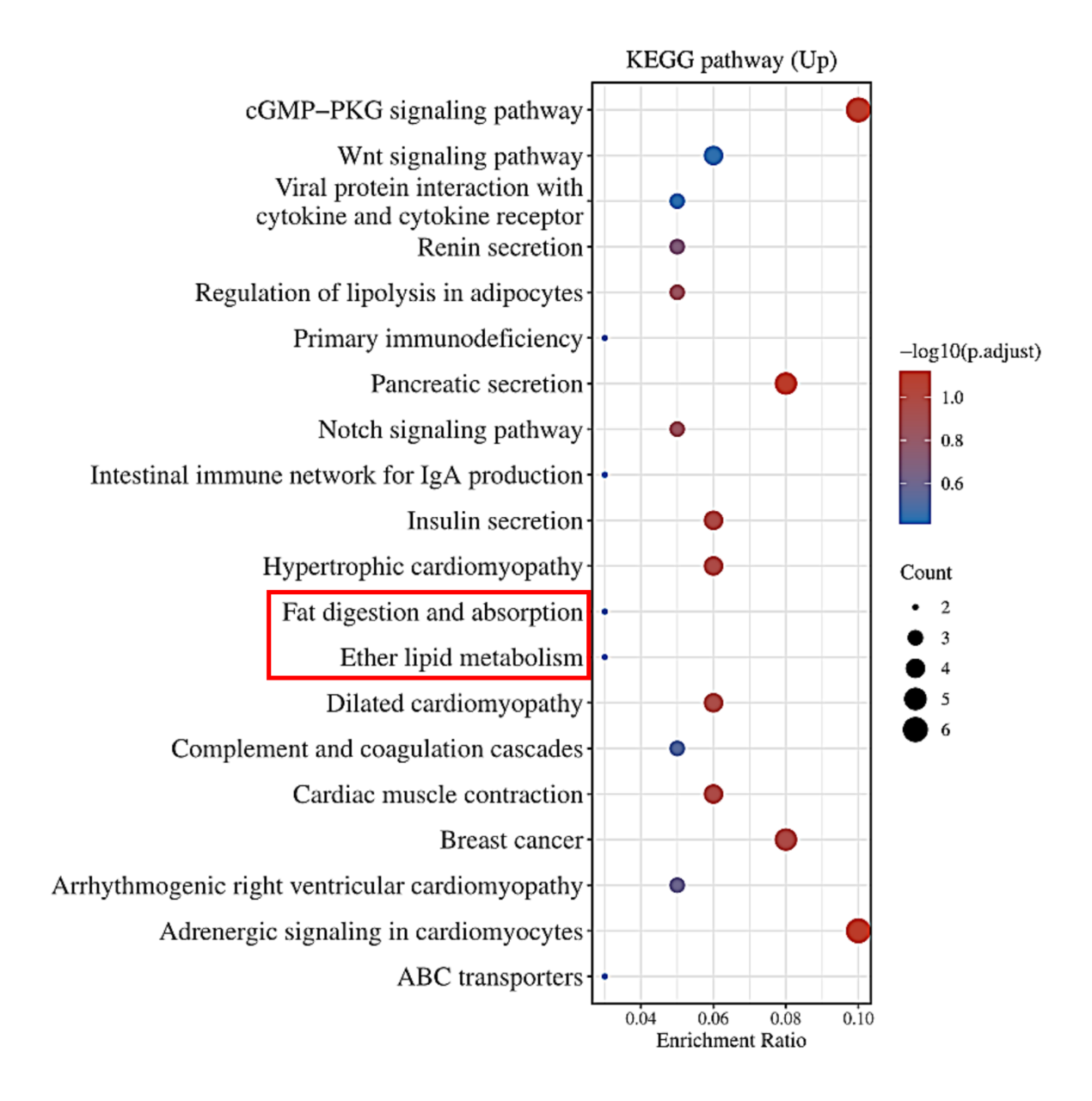


**Fig. S6.** **The transcriptome profiles indicate the role of lipid dysregulation in AAD**

RNA-seq data from the publicly available dataset was analyzed (GSE190635). KEGG analysis of genes up-regulated in aortic tissue from AAD patients compared with HDs. The bubble plot highlights the top 20 most significantly enriched pathways.


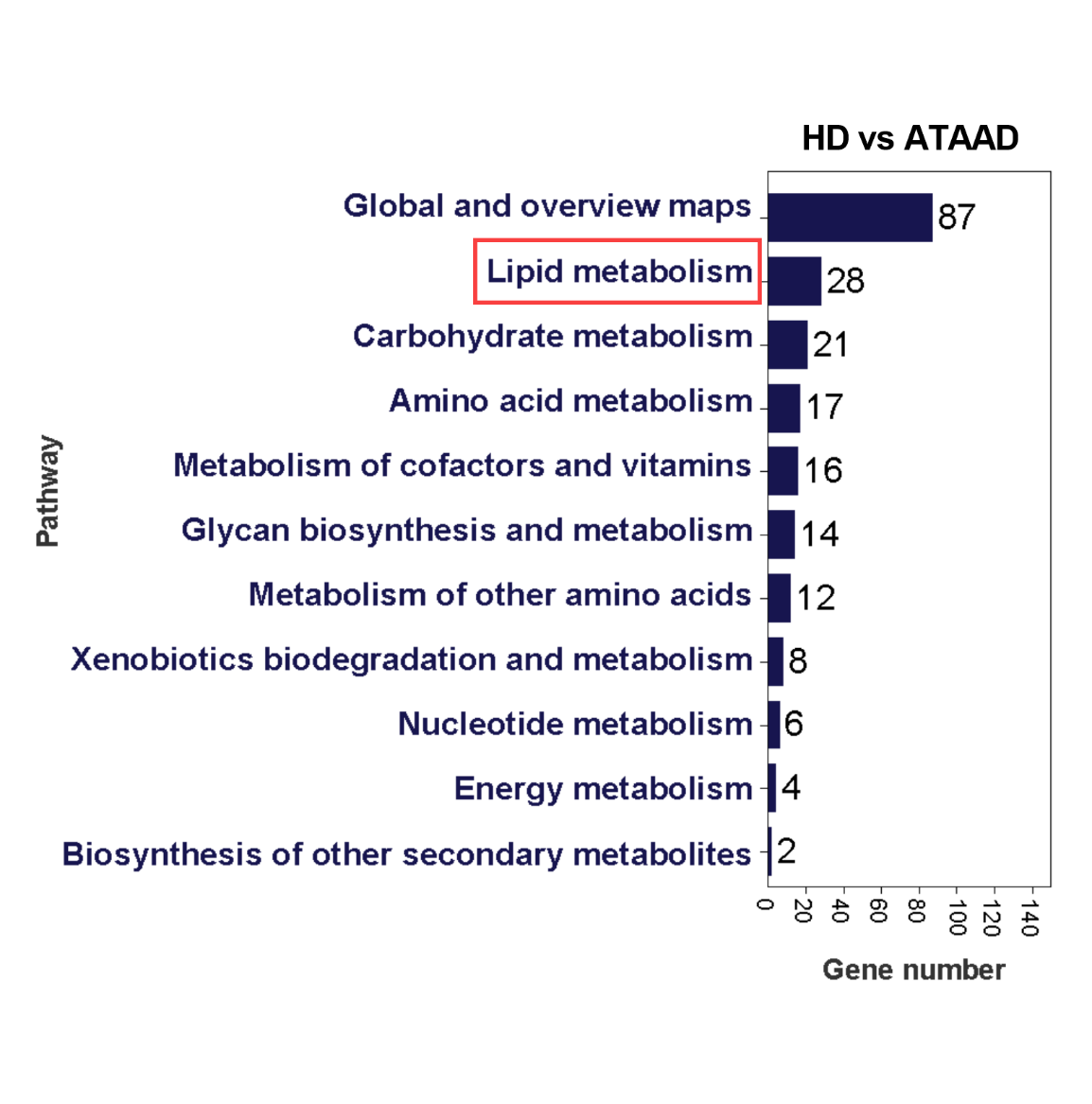


**Fig. S7. The transcriptome profiles indicate lipid dysregulation in CD4^+^ T cells from ATAAD patients**

KEGG analysis focusing on metabolism showed enrichment of DEGs in the lipid metabolism pathway based on RNA-seq in CD4^+^ T cells from ATAAD patients and HDs. ATAAD, type A acute aortic dissection; HD, healthy donors.


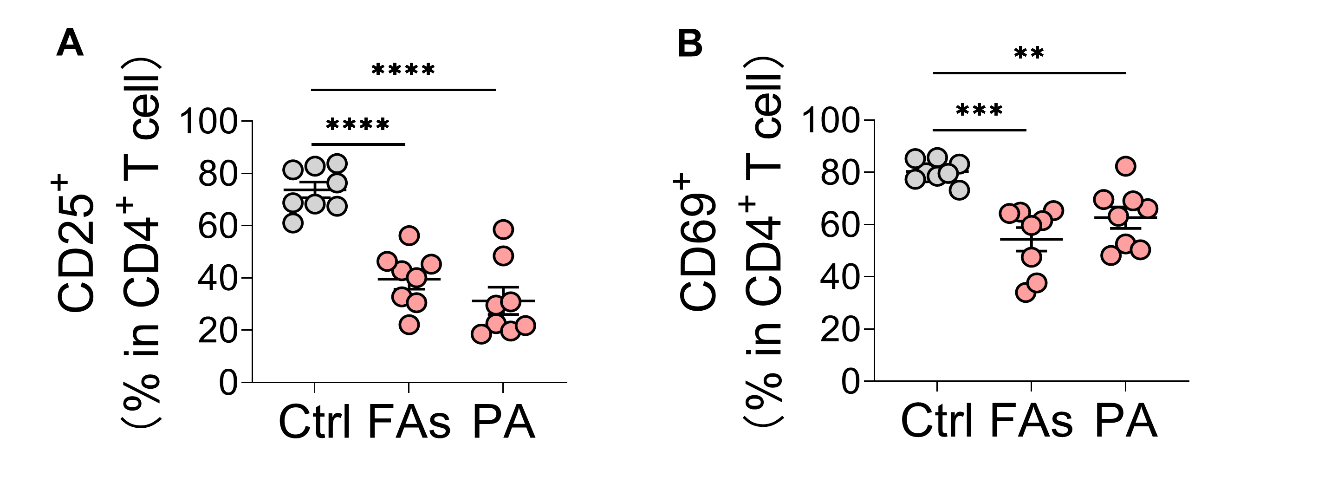


**Fig. S8. FAs and PA inhibit the activity of CD4^+^ T cells in ATAAD**

Flow cytometric quantification was performed to assess the activation percentages of CD4^+^ T cells (CD25^+^and CD69^+^) *in vitro* pretreated or not pretreated with FAs and PA (ATAAD, n=8). One-way ANOVA was performed. *P<0.05; **P<0.01; ***P < 0.001; ****P<0.0001. PA, palmitic acid; FAs, fatty acids.


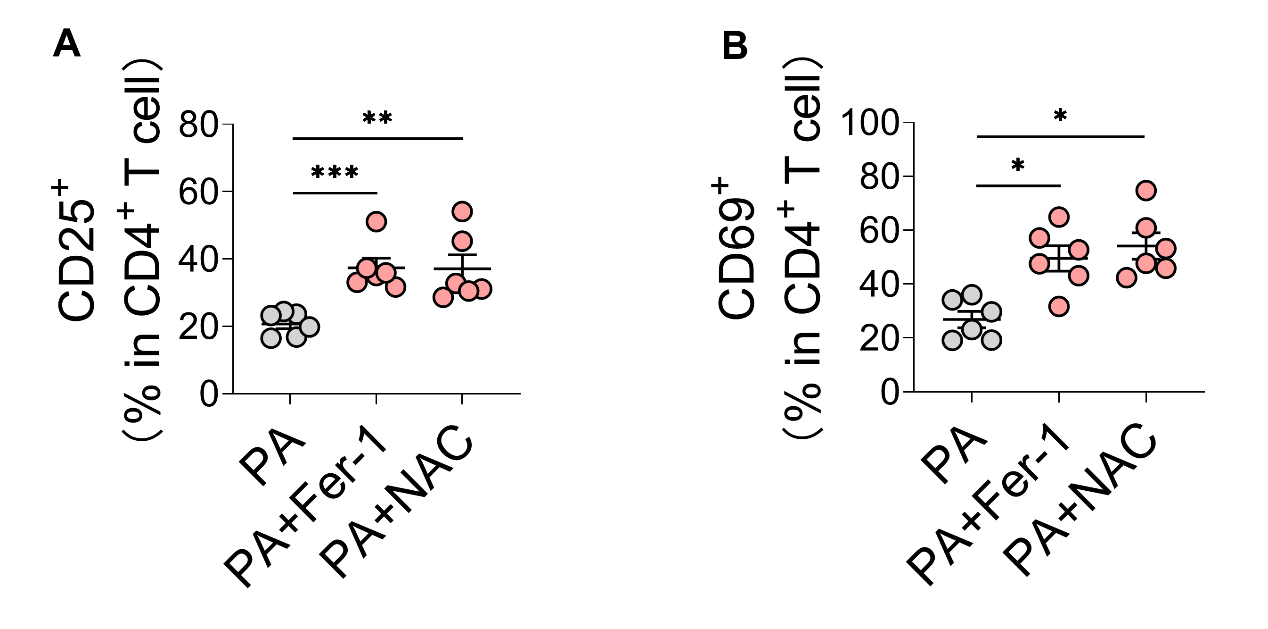


**Fig. S9. Restoration of PA inhibited CD4^+^ T cell activity by ferrostatin-1 and N-acetylcysteine in ATAAD patients**

Activation percentages of CD4^+^ T cells were quantified using flow cytometry. The experimental setup involved pretreatment with ferrostatin-1 and N-acetylcysteine 4 hours before stimulation with PA (ATAAD, n=6). One-way ANOVA was performed. *P<0.05; **P<0.01; ***P < 0.001. PA, palmitic acid; Fer-1, ferrostatin-1; NAC, n-acetylcysteine.


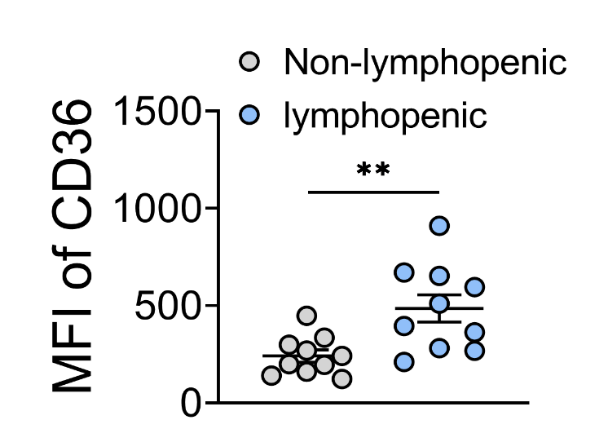


**Fig. S10. CD36 expression on CD4+ T cells from ATAAD patients with or without lymphopenia**CD36 expression was evaluated by flow cytometry in CD4+ T cells from ATAAD patients, grouped as non-lymphopenic (n=10) and lymphopenic (n=10). Unpaired student’s t-tests was performed. *P<0.05; **P<0.01.


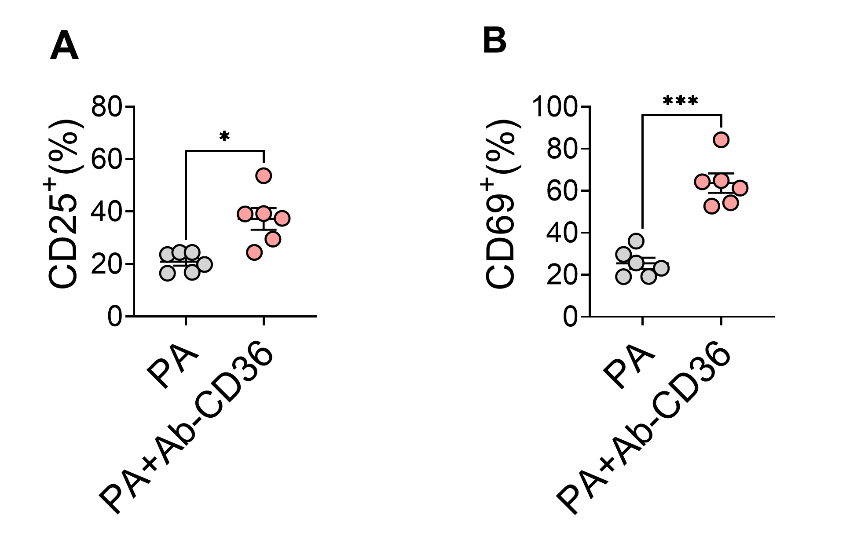


**Fig. S11. Restoration of PA inhibited CD4^+^ T cell activity by blocking CD36 in ATAAD patients**

Activation percentages of CD4^+^ T cells were quantified using flow cytometry. The experimental setup involved pretreatment with CD36 antibody 4 hours before stimulation with FAs or PA (ATAAD, n=6). Paired student’s t-tests was performed. *P<0.05; **P<0.01; ***P < 0.001.
