# Supplementary material for: CD36-mediated ferroptosis destabilizes CD4+ T cell homeostasis in acute Stanford type-A aortic dissection
Source: Cell Death Dis. 2024 Sep 12;15(9):669. doi: 10.1038/s41419-024-07022-9 (PMC11392947; doi:10.1038/s41419-024-07022-9)
Supplement: Supplementary file 2 — Supplementary Tables and Legends [file 41419_2024_7022_MOESM2_ESM.docx]

**Supplemental tables**

**Table S1. Patient characteristics**

| Variables | HD (n=55） | ATAAD（n=55) |
| --- | --- | --- |
| Age, y | 49.3±10.2 | 50.0±11.7 |
| Male | 40(72.7) | 41(74.5) |
| BMI (kg/m²) | 24.9±2.4 | 25.5±3.5 |
| Hypertension | 0(0.0) | 24(43.6) * |
| Coronary heart diseases | 0(0.0) | 1(1.8) |
| Diabetes | 0(0.0) | 0(0.0) |
| Marfan's syndrome | 0(0.0) | 0(0.0) |
| Smoking | 3(5.5) | 4(7.3) |

Data are presented as number (%), mean (SD) or median (IQR).

Abbreviations: ATAAD, Stanford type-A acute aortic dissection; HD, healthy donors; BMI, body mass index. **P* < 0.05 vs. HD

**Table S2. Reagents**

| Antibody | Catalog | Manufacture | Use |
| --- | --- | --- | --- |
| APC/Cyanine7 anti-human CD45 Antibody | 368515 | Biolegend | FCM |
| PE/Cyanine7 anti-human CD3 Antibody | 300419 | Biolegend | FCM |
| PerCP/Cyanine5.5 anti-human CD4 Antibody | 357413 | Biolegend | FCM |
| BV510 anti-human CD8 Antibody | 563256 | BD Pharmingen | FCM |
| APC anti-human CD45RA Antibody | 304111 | Biolegend | FCM |
| PE anti-human CD197 (CCR7) Antibody | 353203 | Biolegend | FCM |
| BV421 anti-Human CD31 Antibody | 564089 | BD Pharmingen | FCM |
| BV421 anti-human CD25 Antibody | 562443 | BD Pharmingen | FCM |
| FITC anti-human CD69 Antibody | 310903 | Biolegend | FCM |
| BV421 anti-human CD36 Antibody | 10009893 | Cayman | FCM |
| BV421 anti-human CD279 Antibody | 367422 | Biolegend | FCM |
| PE anti-human CD36 Antibody | 555455 | BD | FCM |
| PE anti-human CD57 Antibody | B353961 | Biolegend | FCM |
| FITC-AnnexinV | 556419 | BD | FCM |
| PerCP/Cyanine5.5-7AAD | 640936 | Biolegend | FCM |
| Stimulants/protein | | | |
| PHA-P | inh-phap | InvivoGen | Cell culture |
| Other reagents | | | |
| Precision count beads™ | 424902 | Biolegend | FCM |
| RPMI Medium 1640 basic | 8122128 | Gibco | Cell culture |
| CD4 MicroBeads, human | 130-045-101 | Miltenyi Biotec | Cell culture |
| Lymohorep | 07851 | Stemcell | FCM, Cell culture |
| L-Glutamine | A2916801 | Gibco | Cell culture |
| CFSE | C34554 | ThermoFisher | FCM, Cell culture |
| Penicillin-Streptomycin | 15140122 | ThermoFisher | Cell culture |
| Fetal bovine serum | A3160802 | Gibco | Cell culture |
| Phosphate buffered solution | 10010023 | Gibco | Cell culture |
| ROS Detection Kit | 88-5930-74 | ThermoFisher | FCM |
| Mitochondrial membrane potential assay Kit with JC-1 | M34152 | ThermoFisher | FCM |
| Ferrostatin-1 (Fer-1) | S7243 | Selleck | Cell culture |
| MDA assay kit | EEA015 | ThermoFisher | Intracellular MDA detection |
| Iron ion detection | ab83366 | Abcam | Intracellular iron detection |
| N-acetylcysteine (NAC) | S1623 | Selleck | Cell culture |
| Necrostatin-1 (NEC) | S8037 | Selleck | Cell culture |
| Z-VAD-FMK (zVAD) | S7023 | Selleck | Cell culture |
| [Palmic acid](javascript:;) | S3794 | Selleck | Cell culture |
| [Oleic acid](javascript:;)  [Arachidonic acid](javascript:;) | S4707  S6185 | Selleck  Selleck | Cell culture  Cell culture |
| Reverted Aid First Strand cDNA Synthesis Kit | K1622 | ThermoFisher | PCR |
| SYBR Green | K0253 | Thermo Fisher | PCR |
| Trizol | 15596026 | ThermoFisher | Cell Lysis |

Abbreviations: FCM, flow cytometry.

**Table S3. Staining combinations**

| **Staining combinations** |
| --- |
| CD3-PE/Cyanine7；CD4-PerCP/Cyanine5.5; CD8-BV510; CD45-APC/Cyanine7;  CD45RA-APC；CD31-BV421 anti-Human；CD197 (CCR7)-PE |
| CD3-PE/Cyanine7；CD4-PerCP/Cyanine5.5; CD25-BV421; CD69-FITC |
| CD3-PE/Cyanine7；CD4-PerCP/Cyanine5.5; CD279(PD-1)-BV421 |
| CD3-PE/Cyanine7；CD4-PerCP/Cyanine5.5; PerCP/Cyanine5.5-7AAD; FITC-AnnexinV |
| CD3-PE/Cyanine7；CD4-PerCP/Cyanine5.5; FITC-DCF |
| CD3-PE/Cyanine7；CD4-PerCP/Cyanine5.5; PE-CD57 |
| CD3-PE/Cyanine7；CD4-PerCP/Cyanine5.5; PE-CD36 |

**Table S4. Primer sequences for real-time RT-PCR analyses.**

| **Gene** | **Forward primer (5′-3′)** | **Reverse primer (5′-3′)** |
| --- | --- | --- |
| CD36 | CTCTTTCCTGCAGCCCAATG | CTGCCACAGCCAGATTGAGA |
| ACSL1 | CCATGAGCTGTTCCGGTATTT | CCGAAGCCCATAAGCGTGTT |
| STEAP3 | AATGAGAGGCAGGGAGAGC | CTTCAGCCAGAGGTGGGT |
| SAT1 | CCGTGGATTGGCAAGTTATT | TCCAACCCTCTTCACTGGAC |
